# Transcriptome Profiling to Identify Genes Involved in Mesosulfuron-Methyl Resistance in *Alopecurus aequalis*

**DOI:** 10.3389/fpls.2017.01391

**Published:** 2017-08-09

**Authors:** Ning Zhao, Wei Li, Shuang Bai, Wenlei Guo, Guohui Yuan, Fan Wang, Weitang Liu, Jinxin Wang

**Affiliations:** ^1^Key Laboratory of Pesticide Toxicology and Application Technique, College of Plant Protection, Shandong Agricultural University Tai'an, China; ^2^Eco-environment and Plant Protection Research Institute, Shanghai Academy of Agricultural Sciences Shanghai, China

**Keywords:** abiotic stress, acetohydroxyacid synthase, herbicide metabolism, mesosulfuron-methyl, multiple-herbicide resistance, RNA-Seq, shortawn foxtail, weeds

## Abstract

Non-target-site resistance (NTSR) to herbicides is a worldwide concern for weed control. However, as the dominant NTSR mechanism in weeds, metabolic resistance is not yet well-characterized at the genetic level. For this study, we have identified a shortawn foxtail (*Alopecurus aequalis* Sobol.) population displaying both TSR and NTSR to mesosulfuron-methyl and fenoxaprop-*P*-ethyl, yet the molecular basis for this NTSR remains unclear. To investigate the mechanisms of metabolic resistance, an RNA-Seq transcriptome analysis was used to find candidate genes that may confer metabolic resistance to the herbicide mesosulfuron-methyl in this plant population. The RNA-Seq libraries generated 831,846,736 clean reads. The *de novo* transcriptome assembly yielded 95,479 unigenes (averaging 944 bp in length) that were assigned putative annotations. Among these, a total of 29,889 unigenes were assigned to 67 GO terms that contained three main categories, and 14,246 unigenes assigned to 32 predicted KEGG metabolic pathways. Global gene expression was measured using the reads generated from the untreated control (CK), water-only control (WCK), and mesosulfuron-methyl treatment (T) of R and susceptible (S). Contigs that showed expression differences between mesosulfuron-methyl-treated R and S biotypes, and between mesosulfuron-methyl-treated, water-treated and untreated R plants were selected for further quantitative real-time PCR (qRT-PCR) validation analyses. Seventeen contigs were consistently highly expressed in the resistant *A. aequalis* plants, including four cytochrome P450 monooxygenase (CytP450) genes, two glutathione S-transferase (GST) genes, two glucosyltransferase (GT) genes, two ATP-binding cassette (ABC) transporter genes, and seven additional contigs with functional annotations related to oxidation, hydrolysis, and plant stress physiology. These 17 contigs could serve as major candidate genes for contributing to metabolic mesosulfuron-methyl resistance; hence they deserve further functional study. This is the first large-scale transcriptome-sequencing study to identify NTSR genes in *A. aequalis* that uses the Illumina platform. This work demonstrates that NTSR is likely driven by the differences in the expression patterns of a set of genes. The assembled transcriptome data presented here provide a valuable resource for *A. aequalis* biology, and should facilitate the study of herbicide resistance at the molecular level in this and other weed species.

## Introduction

Agrestal weeds reduce crop yields worldwide by 34%, on average, and are thus a considerable threat to food security (Oerke, 2005). Herbicides remain, by far, the most effective tools for weed control. However, weed control failure caused by herbicide resistance is an increasing and critical global problem. Herbicide resistance in weeds is now widely recognized as the consequence of the adaptive evolution of large and genetically variable weed populations to the intensive selection pressure exerted by applied herbicides (Jasieniuk et al., 1996; Powles and Yu, 2010). Because resistant weeds can survive herbicide applications through a variety of mechanisms (Powles and Yu, 2010; Beckie and Tardif, 2012; Délye et al., 2013), a better understanding of these mechanisms is crucial to devise strategies for more efficient weed control.

Currently, the mechanisms of resistance to herbicides may be divided into two broad categories: target-site resistance (TSR) and non-target-site resistance (NTSR). TSR, which arises from the overproduction of the target enzyme or from structural changes in the herbicide binding site, is now well-characterized in weeds (Délye et al., 2013). NTSR arises by any mechanism that reduces the concentration of herbicide reaching the target site (Preston, 2004; Yuan et al., 2007; Powles and Yu, 2010; Délye, 2013; Délye et al., 2013). An enhanced rate of herbicide metabolism (hereafter, “metabolic resistance”) is by far the dominant NTSR mechanism (Yu and Powles, 2014b). It often involves cytochrome P450 monooxygenases (CytP450s), glutathione S-transferases (GSTs), glucosyltransferases (GTs), ATP-binding cassette (ABC) transporters, as well as other enzyme systems, such as oxidases and hydrolases, which can also metabolize herbicides (Preston, 2004; Délye, 2013). Compared with TSR, NTSR is understudied because its mechanisms involve multiple gene activities (Petit et al., 2010a,b). Moreover, NTSR may confer to weeds an unpredictable resistance to herbicides irrespective of their mode of action (Petit et al., 2010b), and this clearly poses a very serious threat to sustainable chemical weed management.

Shortawn foxtail (*Alopecurus aequalis* Sobol.) is a diploid and partly cross-pollinated (c. 40%) winter-annual grass weed that is widely distributed in some overwintering crop regions in China (Guo et al., 2016). This weed has a strong tillering capacity that enhances its competitive ability against wheat (*Triticum aestivum* L.) seedlings, generating yield losses of more than 50% (Tang et al., 1990; Guo et al., 2015a). As a weed, *A. aequalis* is most efficiently controlled by herbicide applications. The excellent efficacy and selectivity of acetyl-CoA carboxylase (ACCase)- and acetohydroxyacid synthase (AHAS)-inhibiting herbicides have led to their widespread adoption for the post-emergence control of *A. aequalis*, but also its subsequent evolution of resistance (Yu and Powles, 2014b). Today, in some regions of China *A. aequalis* has evolved a high level of resistance to many herbicides with different mechanisms of action, such as fenoxaprop-*P*-ethyl and mesosulfuron-methyl. The ACCase gene mutations at Ile1781, Asp2078, or Ile2041, and the AHAS gene mutations at Pro197 or Trp574, were all found to decrease the enzyme sensitivities, the main driver of resistance in *A. aequalis* (Guo et al., 2015b, 2016; Xia et al., 2015). Although an evolved resistance to herbicides is likely due to TSR in many cases, NTSR is now considered the predominant mode of resistance in grass weeds to the ACCase- and AHAS-inhibiting herbicides (Délye, 2013). Moreover, TSR and NTSR can evolve together under the selection pressure exerted by herbicides: hence, both may occur within the same weed species, in the same population, or even in the same individual (Délye et al., 2010; Petit et al., 2010a). Unfortunately, researchers often neglect other coexisting resistance mechanisms after identifying the presence of TSR. Not surprisingly, the NTSR mechanisms in the herbicide-resistant *A. aequalis* have not yet been investigated.

Whole-transcriptome sequencing (RNA-Seq) technology is a powerful tool to unravel the genetic basis of the herbicide stress response in weeds (Unver et al., 2010; An et al., 2014), and to identify the genetic differences between the resistant and sensitive plants, before and after a herbicide application (Metzker, 2010). This exceptionally accurate technology has been applied to many non-model plant species, including weeds (Gaines et al., 2014; Türktaş et al., 2015). Recently, by using RNA-Seq, several genes were identified in weeds that confer NTSR to different herbicides, such as in rye-grass (*Lolium rigidum* Gaudin) (Gaines et al., 2014; Duhoux et al., 2015, 2017), black-grass (*Alopecurus myosuroides* Huds.) (Gardin et al., 2015), American sloughgrass (*Beckmannia syzigachne* Steud.) (Pan et al., 2016), and flixweed (*Descurainia sophia* L.) (Yang et al., 2016). Nevertheless, owing to the different herbicide-use histories and weed species, the genes involved in metabolic resistance might also be different (Délye et al., 2013; Yu and Powles, 2014b). Therefore, most of the genetic mechanisms underpinning NTSR remain largely unknown among different weed species, including *A. aequalis*.

In our prior study an *A. aequalis* population with multiple resistance to mesosulfuron-methyl and fenoxaprop-*P*-ethyl was identified; two amino acid substitutions, Pro197Arg in the AHAS gene and Ile2041Asn in the ACCase gene, were confirmed in the individual plants of this resistant population (Guo et al., 2016). Nevertheless, CytP450s may play an important concurrent role in the fenoxaprop-*P*-ethyl resistance because the CytP450s inhibitor, piperonyl butoxide (PBO), can significantly reduce the GR_50_ (i.e., the herbicide rate causing a 50% growth reduction in plants) (Pan et al., 2015). Cross-resistance patterns were also identified and they indicated the existence of metabolic resistance in the resistant *A. aequalis* population (Guo et al., 2016). In the present study, the mechanism of NTSR to mesosulfuron-methyl was also confirmed by applying the CytP450 inhibitor, malathion. In particular, both an RNA-Seq transcriptome analysis and qRT-PCR experiments were conducted to identify and validate the specific genes involved in NTSR to mesosulfuron-methyl in the *A. aequalis* study population.

## Materials and methods

### Plant materials and growth conditions

The collection and storage of seeds from the resistant (R, AH18-origin) and susceptible (S, SD01-origin) *A. aequalis* populations were described in Guo et al. (2016). The sensitivity of these two populations was tested; a purified R population (AH18) homozygous for the ACCase and AHAS gene mutations and a confirmed S population (SD01) were both acquired (Guo et al., 2016). Prior to their planting, the seeds were germinated as described by Guo et al. (2015a). The pre-germinated seeds were sown in 12-cm-diameter, 11-cm-deep plastic pots (20 seeds per pot) containing moist loam soils. Next, the pots were randomly placed in a controlled greenhouse (natural light, 25/15°C, ~75% relative humidity), where they were watered every other day. For each pot, the weed seedlings that emerged were thinned to 15 that were evenly sized at the two- to three-leaf stage.

### Sensitivity to mesosulfuron-methyl following the Cytp450s inhibitor malathion

Whole-plant dose-response experiments were carried out to determine the GR_50_ values of the purified R (AH18) and confirmed S (SD01) populations to mesosulfuron-methyl in the absence and presence of malathion. The malathion used in this experiment was formulated in a mixture of a commercial solvent and an emulsifier. The mesosulfuron-methyl was applied by using a commercial formulation—30 g L^−1^ oil-miscible flowable concentrate (Bayer, Hangzhou, China)—diluted with distilled water.

When the *A. aequalis* seedlings reached the three- to four-leaf stage they were ready for use in the whole-plant dose-response experiments. These experiments were done twice in a completely randomized design that had three replicates. In each experimental repeat there was a total of 84 pots used (2 malathion rates × 2 populations × 3 replicates × 7 treatments). Malathion was applied as an active ingredient (ai), at a rate of 0 or 1,000 g ai ha^−1^, at 1 h before the herbicides were applied (Preston et al., 1996). Mesosulfuron-methyl application rates of 0, 3, 9, 27, 81, 243, and 729 g ai ha^−1^ were used to treat the R plants, while rates of 0, 0.04, 0.11, 0.33, 1, 3, and 9 g ai ha^−1^ were used on the S plants. These herbicide application rates were adopted to represent the GR_50_ value for each population in the absence of CytP450 inhibitors. The control plants were treated with the mixture (commercial solvent and emulsifier) used to formulate the malathion. All the herbicides were applied by using an air-compressed, moving-nozzle cabinet sprayer which delivered 450 L ha^−1^ of water at a pressure of 0.275 MPa, via a flat-fan nozzle positioned 50 cm above the foliage. After being treated, the experimental plants were returned to the controlled greenhouse. Three weeks later, their aboveground shoots were harvested and oven-dried at 80°C for 72 h and their dry weight data were recorded.

Plant data obtained from the mesosulfuron-methyl dose-responses were expressed in relative terms, as a percentage of the control. The datasets from the replicate experiments were analyzed by ANOVA that used the general linear model procedure in SPSS v.19.0.0 (IBM Corporation, Armonk, USA). Because the variance among the replicated experiments was not significant (*p* > 0.05), the data were pooled and fitted to a non-linear regression analysis in SigmaPlot v.12.0 (Systat Software, Inc., San Jose, USA). The GR_50_ was determined by using a four-parameter log-logistic equation (Seefeldt et al., 1995). The fitted model was as follows:

$$Y=C+\frac{D-C}{\left[1+{\left(\frac{X}{GR_{50}}\right)}^{b}\right]}$$

where *C* is the lower limit of the response, *D* is the upper limit of the response, *X* is the herbicide application rate, and *b* is the slope of the curve at the GR_50_.

To estimate the resistance levels, the resistance index (RI) was calculated as follows:

$$RI=\frac{GR_{50}(R)}{GR_{50}(S)}$$

### Sample collection

Ten seeds were randomly selected from the purified R (AH18) and confirmed S (SD01) populations, respectively, for germination and onward cultivation to maturity. Mature seeds (AH18-F1/SD01-F1) were collected from each individual plant. To study metabolic resistance, one seed from each individual-plant progeny was randomly selected to make up a 10-seed combination. The experimental design included three biological replicates of R and S for the untreated control (CK), water-only control (WCK), and mesosulfuron-methyl (T; 1 × labeled field rate, 9 g ai ha^−1^) treatments, for a total of eighteen 10-seed combinations of the R and S biotype.

The selected 180 seeds were germinated and cultivated to the three- to four-leaf stage under the above-mentioned experimental conditions. Leaf samples for the untreated control were collected at time point 0 (R_CK/S_CK), while those from the water-only control (R_WCK/S_WCK) and the mesosulfuron-methyl treatments (R_T/S_T) were collected 24 h after the treatments were applied. Each sample consisted of a two-leaf mixture: a 4-cm-long, newest emerging leaf, and a 3-cm-long, first fully expanded leaf (both taken from each of the 10 selected plants). A total of 18 samples were thus collected (3 biological replicates × 3 treatments × 2 populations). All of these samples were frozen immediately in liquid nitrogen to avoid RNA degradation and/or induction of plant responses to wounding; they were then pulverized for the total RNA extraction. The remaining seeds (AH18-F1/SD01-F1) from each individual-plant progeny were used in parallel to those for RNA-Seq—they were likewise planted, cultivated to the three- to four-leaf stage, and used as plant materials in the subsequent validation experiment by quantitative real-time reverse transcription polymerase chain reaction (qRT-PCR).

### RNA extraction, cDNA library preparation, and illumina sequencing

The total RNAs from the 18 samples were extracted with Transzol Up (TransGen Biotech, Beijing, China) according to the manufacturer's protocol and they were treated with DNase I (Takara, Beijing, China). Total RNA degradation and contamination were monitored on 1% agarose gels. RNA purity was checked using a NanoPhotometer® spectrophotometer (IMPLEN, West Lake Village, USA), and the RNA concentration was measured using a Qubit RNA Assay Kit and a Qubit 2.0 Fluorometer (Life Technologies, Gaithersburg, USA). The RNA integrity was assessed with a RNA Nano 6000 Assay Kit as part of the Agilent Bio analyzer 2,100 system (Agilent Technologies, Palo Alto, USA). The highest-quality RNA samples were selected for preparing the cDNA library.

The cDNA library construction and the Illumina sequencing were both performed by Annoroad Gene Technology Co., Ltd (Beijing, China). Briefly, the sequencing libraries were generated via a NEBNext Ultra™ RNA Library Prep Kit for Illumina (NEB, Ipswich, USA) following the manufacturer's instructions. A total of 3 μg of RNA per sample was used as the input material for the RNA-sample preparations. Poly (A) mRNA was purified from total RNA by using poly-T oligo-attached magnetic beads, and then fragmented into short sequences. These cleaved RNA fragments were transcribed into first-strand cDNA by a Moloney Murine Leukemia Virus (M-MuLV) Reverse Transcriptase (RNase H-) and by random hexamer primers; this step was followed by a second-strand cDNA synthesis in a reaction buffer which included dNTPs, DNA polymerase I, and RNase H. After end-repair and adapter-ligation, the products were amplified through PCR and purified by using a QIAquick PCR Purification Kit (QIAGEN, Valencia, USA) to construct the cDNA library. The cDNA libraries were sequenced on the Illumina Hiseq™ 4000 platform (Illumina Inc., San Diego, USA) and 150-bp paired-end reads were generated.

### RNA-seq data filtering, *de novo* transcriptome assembly, and gene functional annotation

Raw image data files from the Illumina Hiseq™ 4000 were transformed to raw reads by the Consensus Assessment of Sequence and Variation (CASAVA, v.1.8.2) base recognition program, and then stored in FASTQ-formatted files. Raw reads containing the adapter poly-N (>5% of the unknown sequences designated as “N”) and the low-quality reads (>15% bases with a quality value <20) were filtered out by in-house Perl scripts to obtain high-quality, clean reads. All downstream analyses were based only on these clean reads of high quality.

A *de novo* transcriptome assembly was accomplished in Trinity v.20140717 (Grabherr et al., 2011) under its default parameter values, by which the transcripts and unigenes—the longest transcript of a set of transcripts that appears to originate from the same transcription locus—were obtained. To assess the accuracy of assembly, all of the unigenes were matched to the sequences of an annual model plant, false-brome (*Brachypodium distachyon* [L.] Beauv.), using the BLAST-Like Alignment Tool (BLAST) with *E* ≤ 1e-20. The assembled transcripts were translated into all six possible open reading frames (ORFs) through TransDecoder v.20140717, and their proper translation was defined as the one that gave the longest amino acid sequence (Saha et al., 2002).

To obtain comprehensive, functional information regarding the sequences, gene function was annotated through publicly accessible databases—NCBI non-redundant protein sequences (Nr), NCBI non-redundant nucleotide sequences (Nt), Universal Protein Resource (UniProt), protein family (PFAM), and orthologous groups and functional annotation (eggNOG)—by means of local BLAST programs (NCBI, NIH, Bethesda, USA) which used a significance threshold of *E* < 1e-5. Meanwhile, the software programs HMMER v.3.1b1 (Finn et al., 2011), signalIP v.4.1 (Petersen et al., 2011), and TMHMM v.2.0 (Krogh et al., 2001) in Trinotate were run to identify the protein domains, to predict the signal peptides, and to locate the trans membrane regions, respectively.

Next, the gene ontology (GO, http://www.geneontology.org/) annotation for the unigenes was implemented with Blast2go (Conesa et al., 2005), set to an *E*-value cutoff = 1e-5. The GO functional classifications were performed using WeGO software (http://wego.genomics.org.cn/cgi-bin/wego/index.pl) (Ye et al., 2006). The pathway assignments were carried out by sequence searches against the Kyoto Encyclopedia of Genes and Genomes database (KEGG, http://www.kegg.jp/), which used the KEGG Automatic Annotation Server (KAAS, http://www.genome.jp/kegg/kaas/) set to an *E*-value threshold < 1e-10.

### Identification and analysis of the differentially expressed genes (DEGs)

The assembled transcriptome was used as the reference database, and the clean reads were mapped back onto the reference transcriptome by Bowtie 2 v.2.2.3 (Langmead and Salzberg, 2012). The read count for each gene in each sample was obtained from the mapping results by RSEM (RNA-Seq by Expectation Maximization) (Li and Dewey, 2011); then, the mapped read counts for each transcript were normalized, to eliminate the effects of the sequencing depth and gene length on the gene expression levels, by RPKM (Reads Per Kilobase Millon Mapped Reads) (Mortazavi et al., 2008). In this way, the gene expression levels were determined for each sample and could be directly compared. Next, the differences in the expression abundance of each gene per transcript between sample pairs were calculated by using DESeq 2 v.1.4.5 (Love et al., 2014). Each resulting *p*-value was adjusted to a *q*-value, following the Benjamini-Hochberg procedure for controlling the false discovery rate (Storey and Tibshirani, 2003). Those genes with a *q* < 0.05 and |log2(fold change)| ≥ 1 were identified as DEGs.

Expression differences were compared between the untreated R and untreated S at time point 0 (R_CK and S_CK), between R and S at 24 h after the water control treatment (R_WCK and S_WCK), and between R and S at 24 h after the mesosulfuron-methyl treatment (R_T and S_T). Expression differences were also compared within R and S among the untreated (CK), water-only control (WCK), and mesosulfuron-methyl treatments (T). The DEGs were later analyzed by GO and a KEGG enrichment analysis. Both analyses were implemented with a hyper geometric test (Young et al., 2010), in which the *p*-value was adjusted for multiple comparisons as the *q*-value, and the background data are the genes in the whole genome. Those GO or KEGG terms with a *q* < 0.05 were considered as significantly enriched.

### Selection and qRT-PCR validation of the candidate metabolic resistance contigs

To select candidate contigs for metabolic resistance, three factors were simultaneously considered: their statistical significance, their magnitude of expression differences, and their annotations related to known herbicide metabolism genes and signaling functions. The expression level of each contig was first measured by qRT-PCR in the original RNA samples that were used in the RNA-Seq experiment. For the normalization of gene expression and to ensure the reliability of the qRT-PCR data, the ubiquitin (*UBQ, JN599096.1*) and glyceraldehyde-3-phosphate dehydrogenase genes (*GAPDH, JN599100.1*) were used as the internal control genes (Petit et al., 2012). Gene-specific primers were designed according to the sequences of the selected unigenes in Primer Premier v.5.0 (Premier Biosoft International, Palo Alto, USA). The sequences of these primers are listed in Supplementary Table S4.

The qRT-PCR was performed in 96-well plates on the Bio-Rad CFX96 Touch™ real-time PCR system (Bio-Rad, Richmond, USA), which used the TransStart® Top Green qPCR SuperMix (*TransGen* Biotech, Beijing, China). Each reaction was conducted in a 20-μL mixture—consisting of 10 μL of 2 × TransStart® Top Green qPCR SuperMix, 0.4 μL of forward primer, 0.4 μL of reverse primer, 0.4 μL of cDNA, and 8.8 μL of RNase-free ddH_2_O—with four replicates per cDNA sample. The qRT-PCR programs consisted of a 2-min incubation phase at 95°C, followed by 45 cycles, with each cycle consisting of a 10-s incubation at 95°C, a 15-s incubation at 60°C, and a 20-s incubation at 72°C. At the end of the amplification cycle, melting curve analyses were performed to confirm the specificity of qRT-PCR primers. The amplification efficiencies were derived from a standard curve generated by a 10-fold serial dilution points of cDNA (Pfaffl, 2001). A similar amplification efficacy (87.6–99.7%) for the target and internal control genes was observed. The fold-change in gene expression (as 2^−ΔCt^) was calculated by using the comparative Ct method (Schmittgen and Livak, 2008), relative to the S samples, where ΔCt = [Ct target gene-Ct mean of the two internal control genes]; three biological replicates were obtained. Statistical analysis of the qRT-PCR data was conducted using the Student's *t*-test (*p* < 0.05) procedure of SPSS software.

Furthermore, the expression patterns of those contigs, whose relative expression data provided by qRT-PCR agreed with the RNA-Seq-detected profiles (i.e., the contigs whose expression levels were significantly higher in the R than in the S samples in both the RNA-Seq and qRT-PCR experiments), were also examined in the parallel plant materials—for which the seed germination, plant cultivation, and total RNA extraction followed the above-mentioned methods. Their cDNA was later obtained, and qRT-PCR was carried out using the same primers and procedure as before.

## Results

### Mesosulfuron-methyl dose-response in the absence and presence of the CytP450s inhibitor malathion

**Figure 1:**
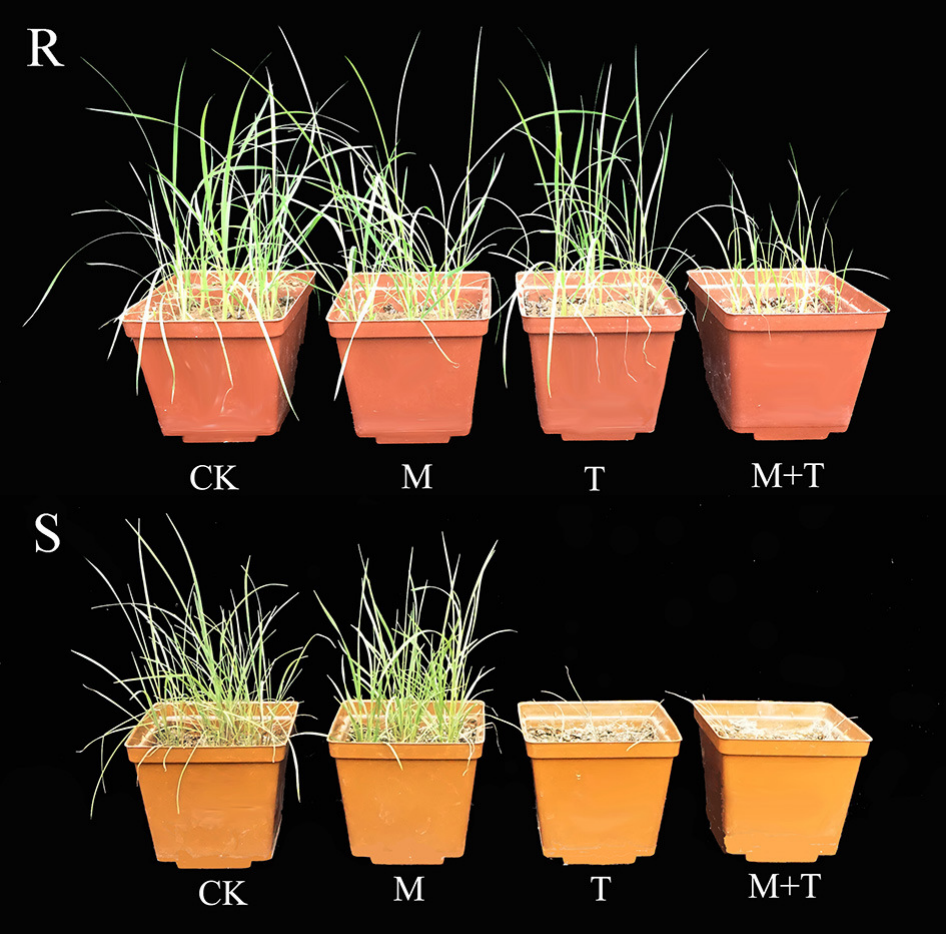
Photographs of R (top) and S (bottom) *Alopecurus aequalis* plants 42 days after treatment, showing the untreated (CK), the malathion-only control (M; 1,000 g ai ha^−1^), the mesosulfuron-methyl treatment (T; 9 g ai ha^−1^), and the effects of the malathion plus mesosulfuron-methyl (M+T; malathion was applied at 1,000 g ai ha^−1^ 1 h prior to the mesosulfuron-methyl application).

**Figure 2:**
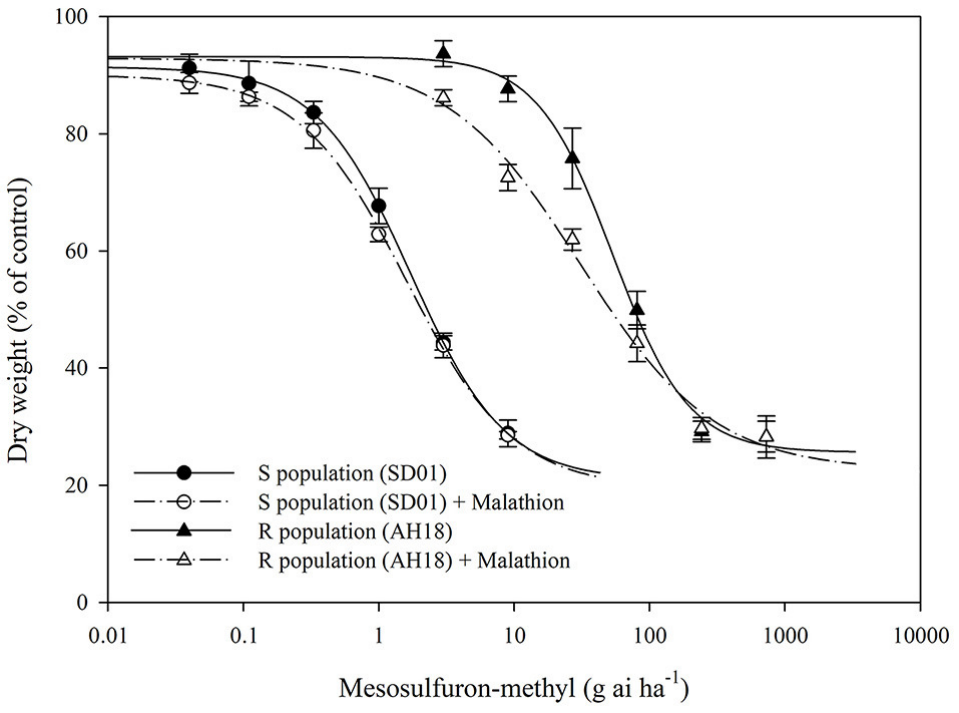
Dose-response curves for the dry weight of the R (AH18) and S (SD01) *Alopecurus aequalis* populations treated with a range of mesosulfuron-methyl doses with (+) or without 1,000 g ai ha^−1^ of malathion. The values are expressed as the percentage of the untreated control. Each data point is the mean ± SE of twice-repeated experiments. The lines were fitted to the mean values using “four-parameter log-logistic equation”.

The whole-plant dose-response experiments demonstrated that the purified R population (AH18) had evolved a high level resistance (RI = 31.15-fold) to mesosulfuron-methyl (Table 1); this was consistent with our previous report (Guo et al., 2016). When malathion alone was applied at 1,000 g ai ha^−1^, there were no visual effects on *A. aequalis* seedling growth, in either the R or S population (Figure 1). Importantly, malathion greatly reduced the resistance level of the R population when it was used before the mesosulfuron-methyl treatment. Under a malathion pretreatment of 1,000 g ai ha^−1^, the GR_50_ value of mesosulfuron-methyl decreased by 41% for the R population (Table 1, Figure 2). By contrast, malathion did not lead to any obvious increase in the susceptibility of the S population to mesosulfuron-methyl, as its GR_50_ decreased little (Table 1). Malathion, the P450 inhibitor, has long been used as an indicator of CytP450 involvement in metabolic resistance to AHAS-inhibiting herbicides. The observation that, in our present study, malathion caused a significant reduction in the resistance level when it was applied with the herbicide indicated the involvement of at least one CytP450 gene in the resistance mechanisms operating in the R biotype.

**Table 1:** The effects of mesosulfuron-methyl on *Alopecurus aequalis* growth with and without malathion pretreatment.

**Treatments**	**GR_50_ values (g ai ha^−1^) (SE)^a^**		**RI^b^**
	**R (AH18)**	**S (SD01)**	
Mesosulfuron-methyl	53.57 (4.34)	1.72 (0.08)	31.15
Mesosulfuron-methyl + malathion (1,000 g ai ha^−1^)	31.12 (1.71)	1.61 (0.19)	18.09

a *GR_50_, the herbicide rate causing a 50% growth reduction of plants. Standard errors are in parentheses*.

b *Resistance levels were indicated by the resistance index (RI). RI = GR_50_ (R)/GR_50_ (S)*.

### Illumina sequencing and *de novo* assembly

**Figure 3:**
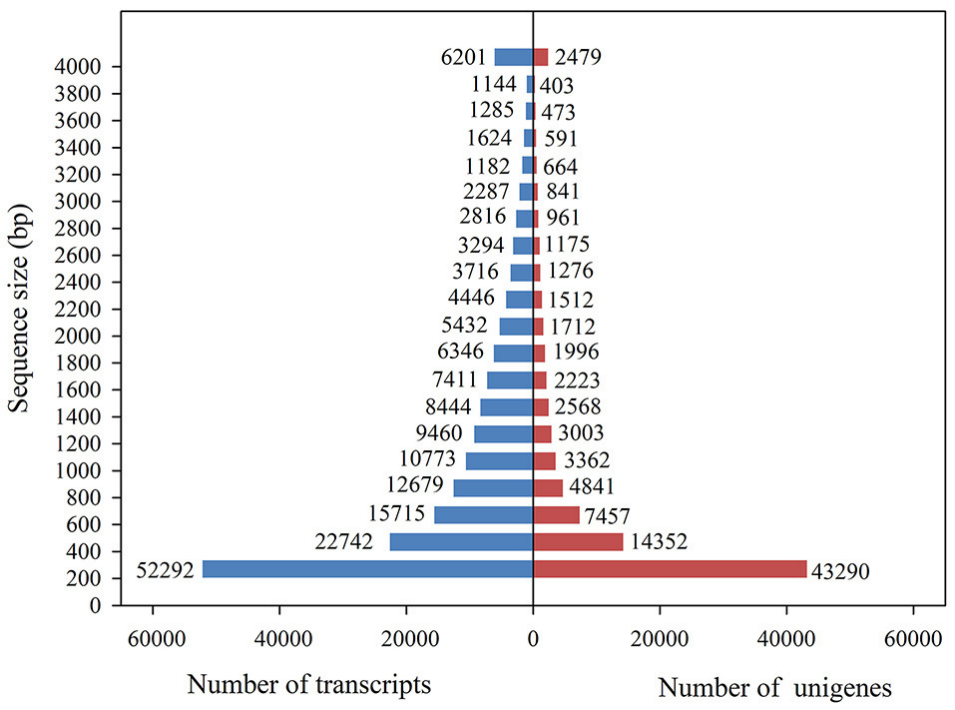
Overview of the transcriptome assembly data for *Alopecurus aequalis*, showing the size frequency distributions of the transcripts and unigenes.

Using the Illumina sequencing technology, a total of 975,586,352 raw sequencing reads were generated from the 18 RNA libraries (S_CK, R_CK, S_WCK, R_WCK, S_T, and W_T; each had three biological replicates). After the quality control and data cleaning, there were 831,846,736 clean reads left over, ranging from 49,270,938 to 60,784,678 per sample, for use in the *de novo* assembly (Table 2, Supplementary Table S1). The clean reads assembled to 179,989 transcripts, with an average length of 1,233 bp. Figure 3 shows the length distribution of these transcripts. Of the 831,846,736 clean reads sequenced, we were able to map the great majority of them (93.02%) back onto the transcripts using Bowtie 2. Among these transcripts, 43,290 unigenes >200 bp and 14,352 unigenes >500 bp, with an overall mean length of 944 bp and an N50 length of 1,857 bp, were obtained by using the longest transcript at each locus of each gene. The length distribution of these unigenes is also shown in Figure 3. *B. distachyon* is now intensively utilized as a model grass species in various biological studies (Draper et al., 2001). By using the BLAST search, 34.40% of the unigenes were successfully matched to the sequences of *B. distachyon*, at an average accuracy of 84.18%.

**Table 2:** Summary statistics of *Alopecurus aequali* transcriptome sequencing and assembly.

**Assembly quality parameters**	
Raw reads number	975,586,352
Clean reads number	831,846,736
Total assembled transcripts	179,989
Total assembled unigenes	95,479
Total nucleotides of unigenes	90,099,088
Maximum length (bp)	131,139
Minimum length (bp)	201
Average length (bp)	944
No. unigenes >200 bp	43,290
No. unigenes >500 bp	14,352
N50 size (bp)	1,857
N90 size (bp)	335

### Gene functional annotation and classification

**Figure 4:**
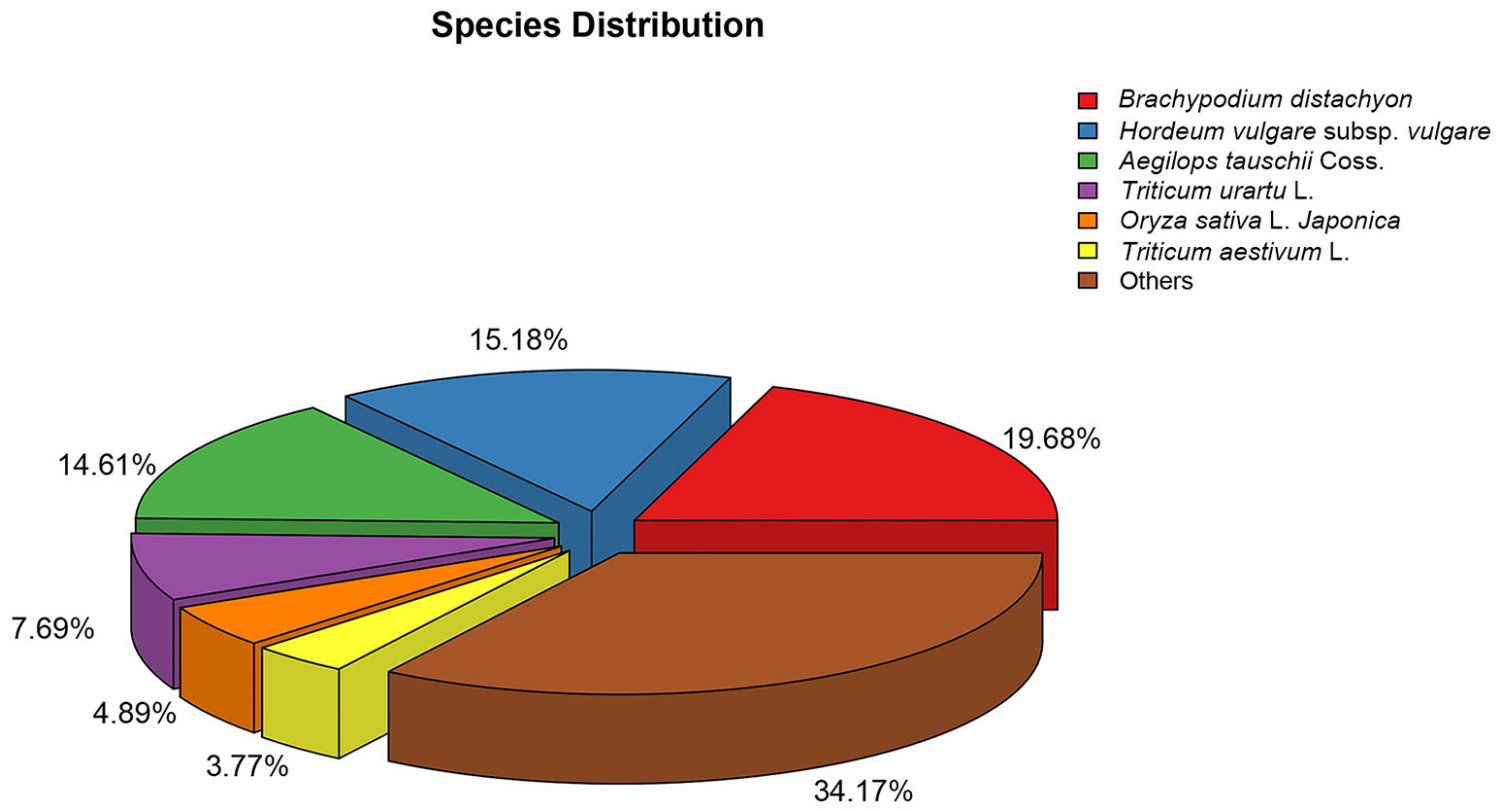
Species distributions of the BLASTX matches of the *Alopecurus aequalis* transcriptome unigenes.

The numbers of unigenes annotated by the databases are summarized in Table 3. Of the 95,479 assembled unigenes, over half were annotated in at least one of the seven databases, whereas <5% had annotations in all seven databases, and the greatest sequence similarity was obtained from the Nr database (Table 3). Based on the Nr database annotations, the unigene sequences of our study species, *A. aequalis*, were most similar to the gene sequences from *B. distachyon, Hordeum vulgare* subsp. *vulgare, Aegilops tauschii* Coss.*, Triticum Urartu* L., *Oryza sativa* L. *Japonica*, and *Triticum aestivum* L. (Figure 4).

**Table 3:** Sequence annotation of the *Alopecurus aequalis* transcriptome.

**Public database**	**Number of unigenes**	**Percentage**
Annotated in NR	44,005	46.09
Annotated in NT	27,974	29.30
Annotated in PFAM	23,078	24.17
Annotated in eggNOG	15,826	16.58
Annotated in GO	29,889	31.30
Annotated in KEGG	14,246	14.92
Annotated in UniProt	35,562	37.25
Annotated in at least one database	53,313	55.84
Annotated in all databases	3,147	3.30

**Figure 5:**
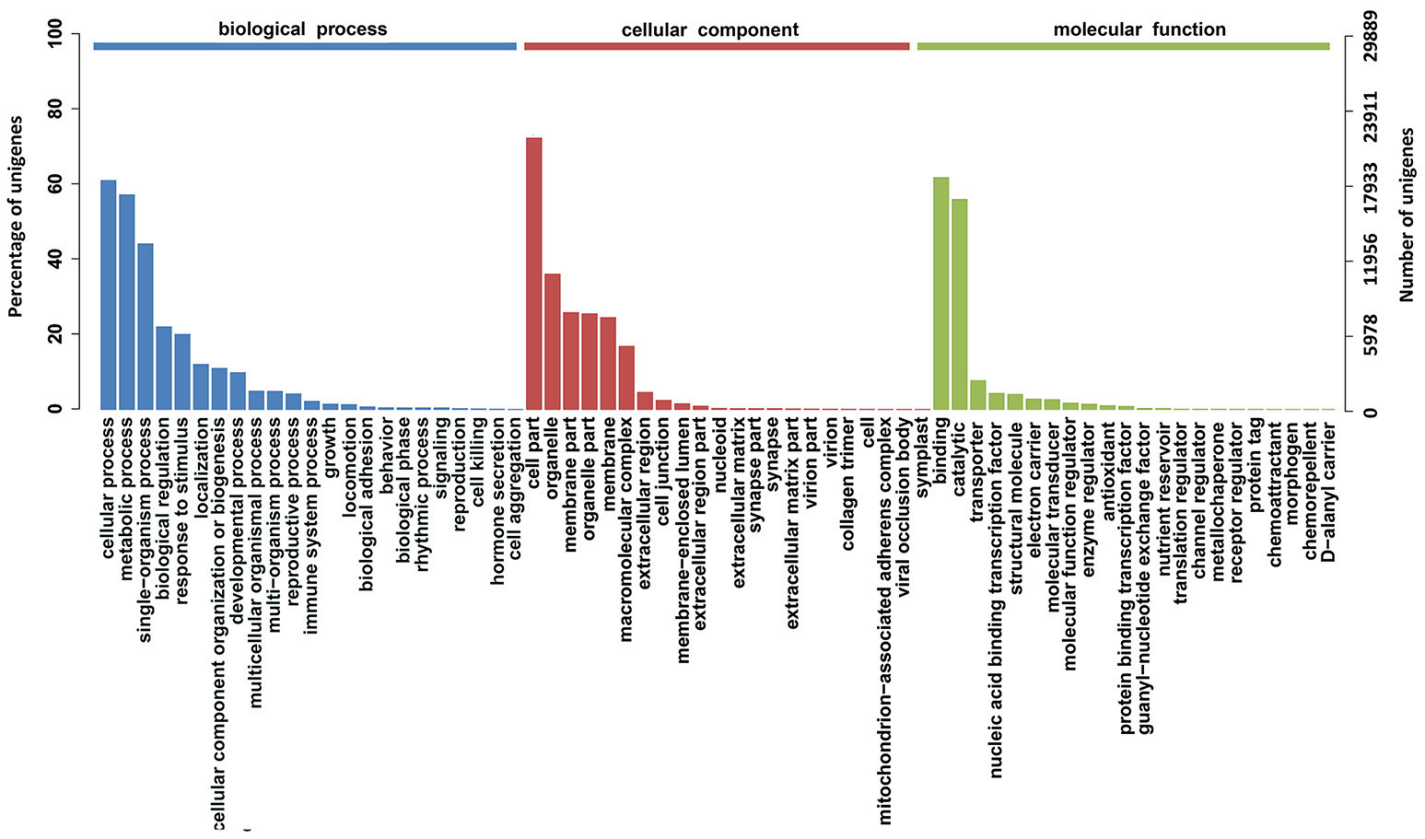
GO function classification of the annotated unigenes in *Alopecurus aequalis*. The unigenes were allocated to three categories: biological process, cellular component, and molecular function.

**Figure 6:**
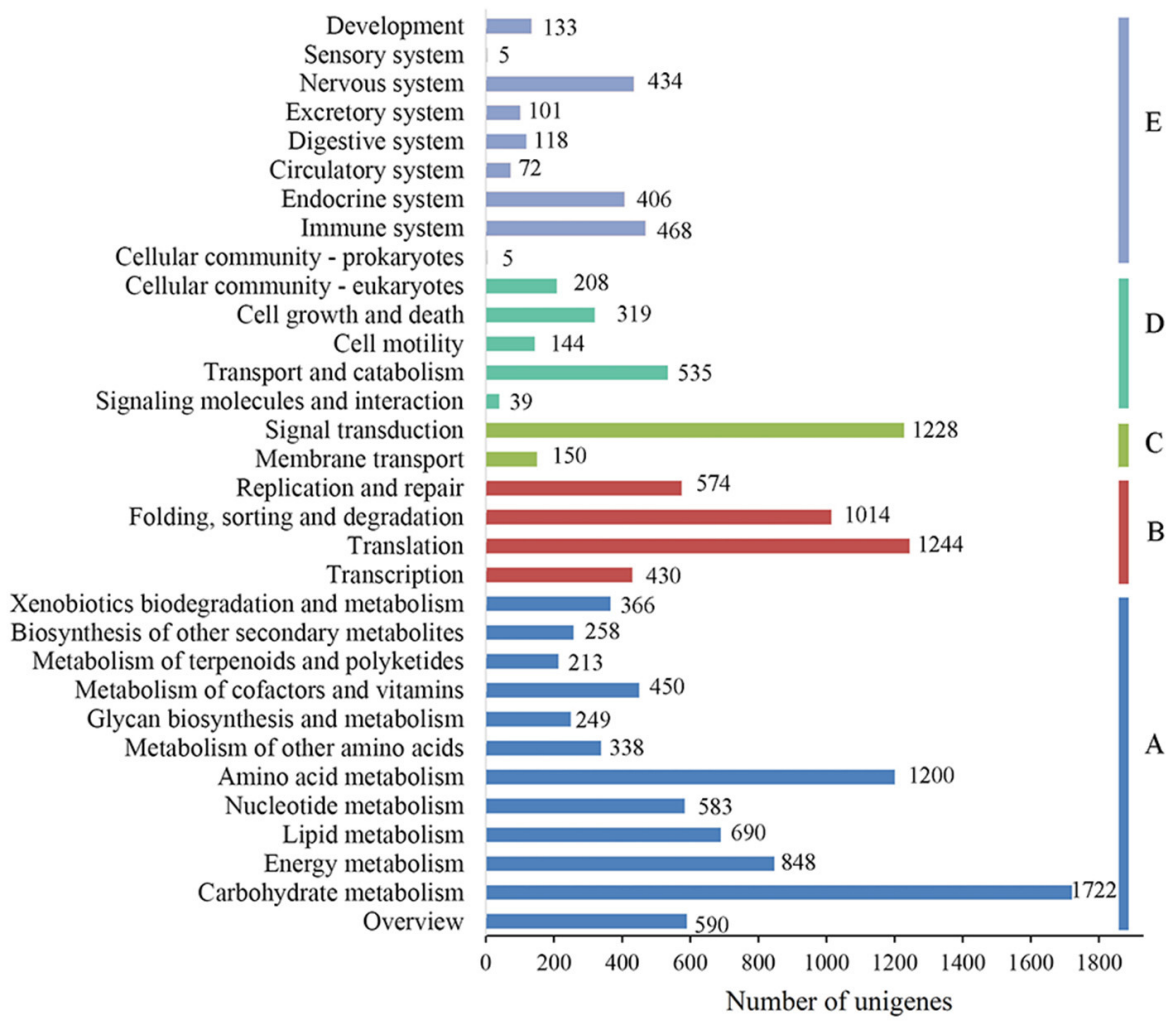
KEGG function classification results of the annotated unigenes in *Alopecurus aequalis*. The y-axis lists the various KEGG pathways. The x-axis indicates the number of genes. According to their participation in these KEGG pathways, the unigenes were divided into five color-coded groups: metabolism **(A)**, genetic information processing **(B)**, environmental information processing **(C)**, cellular processes **(D)**, and organismal systems **(E)**.

To characterize the functional classifications of the annotated unigenes, GO and KEGG analyses were performed to access the distributions of functional categories. A total of 29,889 (29.29%) unigenes were annotated by the GO database; these were classified into 67 functional subgroups consisting of 23 subgroups as “biological process” (BP), 22 as “cellular component” (CC), and 22 as “molecular function” (MF) (Figure 5). In the BP category, cellular process (18,218, 60.95%) and metabolic process (17,088, 57.17%) represented the two major contributors. In the molecular function category, binding (18,469, 61.79%) and catalytic activity (16,732, 55.98%) represented the major contributors. In addition, 14,246 unigenes were assigned to 32 KEGG pathways; these unigenes were primarily involved in “carbohydrate metabolism” (1,722, 12.09%), “translation” (1,244, 8.73%), “signal transduction” (1,228, 8.62%), and “amino acid metabolism” (1,200, 8.42%)” (Figure 6).

### Differential gene expression and functional analysis

**Figure 7:**
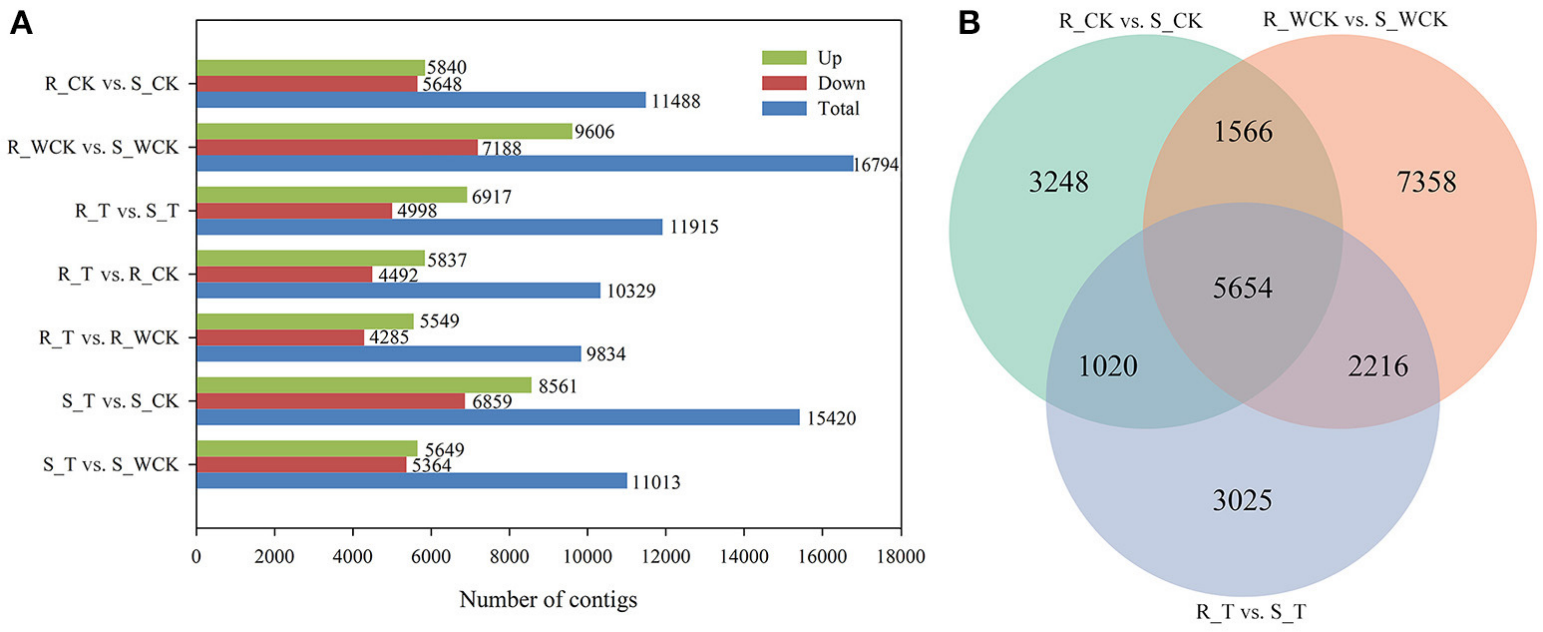
The statistics of the DEGs between the *Alopecurus aequalis* treatment groups. **(A)** The number of DEGs between the different groups. **(B)** Venn diagram showing the number of DEGs between R (AH18) and S (SD01) in the three treatment comparisons.

Constitutive differential expression (i.e., *q* < 0.05 and |log_2_(fold change)|≥1) was apparent between the untreated R and S samples, such that 5,840 contigs were constitutively up-regulated in R, and 5,648 contigs were up-regulated in S (Figure 7A). Differential expression between R and S in all three treatments was evident for 5,654 contigs (Figure 7B). When comparing the mesosulfuron-methyl treatment with the water-only control, a total of 5,549 contigs were up-regulated in R, and 5,649 contigs were up-regulated in S. However, considering only those contigs up-regulated by the mesosulfuron-methyl treatment revealed an overlap of 409 contigs between the R and S populations, including contigs putatively annotated as 10 CYPs, 1 GST, 4 GTs, and 2 ABC transporters (Supplementary Table S2). DEGs between the mesosulfuron-methyl treatments and the untreated control were also identified: there were 5,837 contigs up-regulated and 4,492 contigs down-regulated in R_T relative to R_CK, as well as 8,561 contigs up-regulated and 6,859 contigs down-regulated in S_T relative to S_CK (Figure 7A).

To further characterize the function of the DEGs, we conducted GO enrichment analyses. In total, there were 11,915 DEGs found between the R_T and S_T samples; these were classified into 57 functional groups, namely 22 groups as BP, 17 groups as CC, and 18 groups as MF (Supplementary Figure S1). Among these DEGs, the GO terms of “cellular process” (1587, 13.32%), “metabolic process” (1200, 12.59%), and “single-organism process” (1202, 10.09%)” in the BP category, “cell part” (1912, 16.05%)” in the CC category, and those of “binding” (1691, 14.19%)” and “catalytic” (1691, 13.00%) in the MF category, were all significantly enriched between the R and S population samples.

We also conducted a detailed KEGG enrichment analysis of the DEGs between the R and S populations for each of the three treatments (Supplementary Figures S2–S4). The DEGs between the mesosulfuron-methyl-treated R and S samples were significantly enriched in the 12 pathways (Table 4). Specifically, 17 up-regulated genes were enriched in the “metabolism of xenobiotics by CytP450” pathway, and 16 up-regulated genes were enriched in the “drug metabolizing-cytochrome CytP450” pathway. Notably, the genes involved in the “metabolism by cytochrome CytP450” changed significantly in the KEGG pathway analysis; this result suggested that the CytP450 genes could play a key role in the metabolic resistance to mesosulfuron-methyl in *A. aequalis*.

**Table 4:** The 12 enriched KEGG pathway terms of the DEGs between mesosulfuron-methyl treated R (AH18) and S (SD01) *Alopecurus aequalis* populations.

**KEGG pathway term**	**Map ID**	**Count^a^**		**Background genes^b^**	***q*-value^c^**
		**Up**	**Down**		
Oxidative phosphorylation	map00190	81	1	367	1.46E-07
Plant-pathogen interaction	map04626	39	38	333	1.46E-07
Phenylpropanoid biosynthesis	map00940	26	9	173	1.57E-02
Cardiac muscle contraction	map04260	20	0	64	1.06E-03
Drug metabolism-cytochrome CytP450	map00982	17	7	91	2.32E-03
Cutin, suberine and wax biosynthesis	map00073	17	0	51	1.48E-03
Metabolism of xenobiotics by cytochrome CytP450	map00980	16	7	85	2.32E-03
Chemical carcinogenesis	map05204	16	6	74	1.06E-03
Zeatin biosynthesis	map00908	11	2	25	7.62E-05
Stilbenoid, diarylheptanoid and gingerol biosynthesis	map00945	6	2	20	2.61E-02
Gastric acid secretion	map04971	5	0	8	2.26E-02
Flavonoid biosynthesis	map00941	5	3	18	1.49E-02

a *Number of up- and down-regulated genes enriched in this pathway*.

b *Number of unigenes annotated in this pathway*.

c *The p-value adjusted by the multiple comparisons and expressed here as the q-value*.

### Selection of candidate metabolic resistance contigs

Considering the contigs differentially expressed between the R and S populations, those having predicted annotations related to metabolism and signaling pathways are more likely to be involved in conferring metabolic mesosulfuron-methyl resistance (Gaines et al., 2014). Therefore, we selected those overlapping genes up-regulated in all three comparative groups—i.e., R_T relative to S_T, R_T relative to R_WCK, and R_T relative to R_CK—which had such predicted annotations (Supplementary Table S2). We also selected those up-regulated genes annotated as related to metabolism that had a significantly different expression between R and S in all three treatments (Supplementary Table S3). In particular, the overlapping genes having |log2(fold change)| values in R_T relative to S_T exceeding those of the other two comparable groups (i.e., R_WCK vs. S_WCK and R_CK vs. S_CK) were identified (Supplementary Table S3). In addition, contigs were also chosen that had predicted annotations related to oxidation (e.g., oxidase and peroxidase), hydrolysis (e.g., esterase and hydrolase), and plant stress physiology (e.g., response to plant hormone stimulus) in the above-mentioned comparative groups, along with those contigs with the largest fold-change expression differences but of an unknown function (i.e., no assigned annotation). Based on these criteria, a total of 31 contigs were selected as candidate genes that might confer mesosulfuron-methyl resistance to *A. aequalis* (Table 5). Of these contigs, eight were annotated to the CytP450 family, five to the GST family, five to GT family, and two to the ABC transporter family, with the remainder (11) annotated to other families.

**Table 5:** Selection and identification of the up-regulated contigs annotated and related to metabolic resistance in *Alopecurus aequalis* via RNA-Seq and qRT-PCRs (2^−ΔCt^).

**Gene ID**	**PFAM ID**	**Function annotation^a^**	**Accession number^b^**	**RNA-Seq**		**Fold change: qRT-PCR (2^−ΔCt^)**	
				***q*-value^c^**	**Fold change (R_T/S_T)**	**RNA-Seq samples (R_T/S_T)**	**Parallel materials (R_T/S_T)**
c42830_g1	PF00067.17	CYP71C2	NA	2.78E-19	4.57	2.56^*^^d^	1.96
c39857_g2	PF00067.17	CYP94A2	XP_003578905.1	7.13E-05	12.17	10.00^*^	16.88^*^
c46166_g6	PF00067.17	CYP71D10	XP_003581127.1	1.34E-11	29.59	1.60	0.96
c50769_g1	PF00067.17	CYP71D8	XP_003570845.1	5.58E-03	7.31	2.00^*^	1.01
c21190_g1	PF00067.17	CYP94A1	XP_014753490.1	3.89E-73	23.21	16.67^*^	12.49^*^
c43350_g3	PF00067.17	CYP71A4	XP_003568112.1	3.12E-25	7.82	5.69^*^	32.64^*^
c45454_g1	PF00067.17	CYP734A6	XP_003562608.1	1.63E-08	10.11	9.09^*^	9.89^*^
c46602_g8	PF00067.17	CYP86B1	XP_003573623.1	9.89E-05	3.21	1.51	1.33
c21481_g1	.	GST-T3	XP_010237543.1	3.49E-18	116.54	2.31^*^	1.52
c46293_g5	PF14303.1	GST-T3	KQK05754.1	2.17E-34	90.25	2.33^*^	4.89^*^
c42028_g1	PF13417.1	GST-Z2	XP_003578780.1	2.73E-138	8.96	7.14^*^	1.56
c35468_g1	PF13410.1	GST-Z2	NA	8.96E-37	110.33	2.35^*^	1.22
c45520_g1	PF00043.20	GST-F1	XP_003567701.1	7.64E-11	4.27	7.92^*^	3.25^*^
c49096_g1	.	GT92A1	XP_003581400.3	1.30E-05	5.70	0.97	0.69
c44063_g7	PF00201.13	GT75D1	XP_003571494.1	2.10E-09	8.70	4.56^*^	7.70^*^
c26389_g1	PF00201.13	GT83A1	XP_003559280.2	1.27E-09	44.77	12.21^*^	20.54^*^
c45451_g12	.	GT73B5	NA	8.77E-13	4.81	1.35	1.11
c26062_g1	PF00201.13	GT73C5	XP_003569538.3	3.36E-12	2.84	1.42	0.56
c34002_g1	PF00664.18	ABCC8	XP_010231619.2	2.15E-19	13.12	10.13^*^	6.57^*^
c39076_g1	PF00664.18	ABCB11	NA	3.63E-11	2.61	3.10^*^	4.21^*^
c38775_g3	PF00141.18	Peroxidase	XP_003580878.1	2.59E-12	21.47	2.66^*^	1.03
c38555_g1	PF00724.15	NADH oxidase	XP_003564097.1	3.31E-20	20.77	8.42^*^	6.64^*^
c33496_g2	PF00724.15	NADH oxidase	XP_003557664.1	6.62E-24	151.98	7.89^*^	12.95^*^
c33496_g1	PF00724.15	NADH oxidase	XP_003568938.1	3.32E-41	200.25	24.54^*^	14.14^*^
c41309_g5	PF00135.23	Carboxylesterase	KQK14951.1	1.06E-08	11.40	0.65	0.42
c43601_g1	PF02230.11	Phospholipase	XP_003567798.1	2.97E-03	3.91	0.61	0.55
c48254_g2	PF01546.23	Hydrolase	XP_010231929.1	8.49E-135	6.82	3.54^*^	5.89^*^
c36906_g1	PF03641.9	Decarboxylase	XP_010236986.1	2.38E-108	27.85	2.23^*^	1.37
c39903_g1	PF13419.1	Hydrolase	XP_003558239.1	2.09E-32	11.79	4.52^*^	6.86^*^
c46657_g2	PF13947.1	Kinase	XP_010228098.1	1.14E-42	13.13	7.45^*^	5.22^*^
c47808_g1	PF13947.1	Kinase	XP_014757032.1	4.59E-14	82.34	32.18^*^	28.67^*^

a *The subfamily classification of CytP450 genes was annotated from NCBI non-redundant protein sequences (Nr) database*.

b *Accession number from GenBank of the closest homolog in Brachypodium distachyon. NA, not available because there was no homolog found in B. distachyon for the corresponding gene*.

c *The resulting p-value was adjusted and expressed as the q-value by the Benjamini-Hochberg procedure for controlling the false discovery rate*.

d p < 0.05 is indicated by

* *from the SPSS analysis*.

### qRT-PCR validation of the candidate metabolic resistance contigs

The expression levels of the 31 candidate contigs were first validated by qRT-PCR using the original RNA samples (i.e., R_T and S_T). The relative expression data provided by the qRT-PCR showed that 24 of the 31 candidate contigs exhibited significantly higher expression levels in the R (AH18) than in the S (SD01) population samples (Table 5).

**Figure 8:**
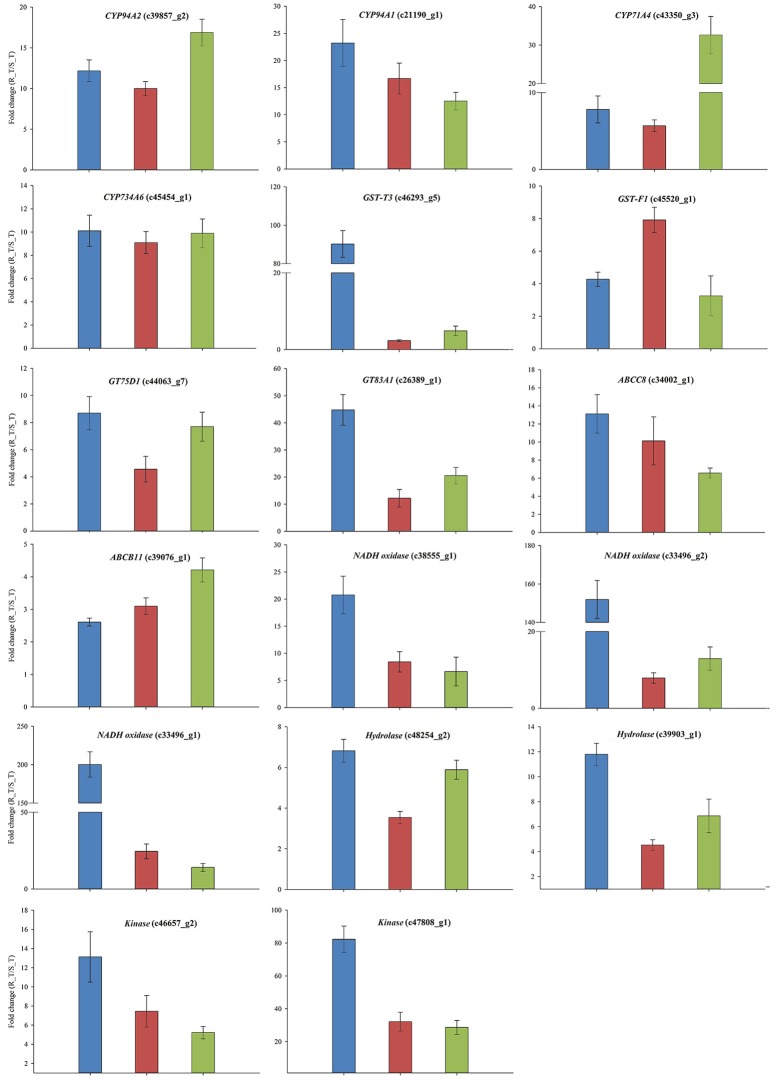
The qRT-PCR validations of the 17 genes that had a consistently up-regulated expression in the R *Alopecurus aequalis* samples. Blue bars represent the RNA-Seq; red bars represent the qRT-PCR validation that used the RNA-Seq samples; and green bars represent the qRT-PCR validation that used the parallel plant materials. Both *UBQ* and *GAPDH* were used as the internal control genes. Means and their SEs from three biological replicates are shown.

The expression patterns of the 24 candidate contigs were evaluated further using the parallel *A. aequalis* plant materials. These parallel samples had a genetic background identical to the RNA-Seq samples. Based on the results of the RNA-Seq and the two rounds of the qRT-PCR validations, a total of 17 candidate contigs were expressed significantly higher in the R (AH18) than in the S (SD01) population samples (Table 5, Figure 8, Supplementary Figure S5). These 17 contigs possibly encoded proteins with homology to four CytP450s (CYP94A1, CYP94A2, CYP71A4, and CYP734A6), two GSTs (GST-T3 and GST-F1), two GTs (GT75D1 and GT83A1), two ABC transporters (ABCC8 and ABCB11), three oxidases, two hydrolases, and two kinases. Although some variation among individual plants was observed, the average expression levels of the candidate contigs provided by qRT-PCR were consistent with the RNA-Seq expression data, thus revealing little deviation in the fold change (Figure 8).

## Discussion

This study aimed to identify the genes present in *A. aequalis* in response to mesosulfuron-methyl, so as to establish a transcriptomic resource for subsequent studies. Our previous study confirmed that the R *A. aequalis* population (AH18) has evolved a high level of resistance to fenoxaprop-*P*-ethyl and mesosulfuron-methyl. Two amino acid substitutions, Ile2041Asn in the *ACCase* gene and Pro197Arg in the *AHAS* gene, were identified in the individual plants of the R population. Furthermore, the NTSR mechanism may take part in the resistance to fenoxaprop-*P*-ethyl by *A. aequalis*. (Guo et al., 2016). Here, we demonstrated that the resistance to mesosulfuron-methyl of the R *A. aequalis* population is also partly due to a NTSR mechanism, because the use of malathion largely reversed this resistance (Table 1, Figures 1, 2). It is well-established that malathion, a cytochrome P450 inhibitor, is able to inhibit the metabolism of the sulfonylurea herbicide, thus reversing a metabolism-based resistance (Christopher et al., 1994; Yu and Powles, 2014a). Similarly, malathion has been used recently to examine metabolism-based resistance to the AHAS-inhibiting herbicide tribenuron-methyl in a resistant *D. sophia* population (Yang et al., 2016). Together, these results support the hypothesis that NTSR co-exists with TSR in resistant plants, as was clearly demonstrated for herbicide-resistant *Lolium rigidum* populations (Han et al., 2016).

Additionally, the area from which the R *A. aequalis* population was collected had received the fenoxaprop-*P*-ethyl application 7–8 years earlier than that of mesosulfuron-methyl. The ACCase-inhibiting herbicide, fenoxaprop-*P*-ethyl, probably “pre-selected” for NTSR to the AHAS-inhibiting herbicide mesosulfuron-methyl (Délye et al., 2011). The underlying genes of the R population involved in metabolism-based resistance, as triggered by mesosulfuron-methyl, are likely also playing an important role in the NTSR to fenoxaprop-*P*-ethyl. Therefore, a RNA-Seq study was warranted for the R *A. aequalis* population (AH18) to better understand metabolic resistance evolution in this weed species.

RNA-Seq technology is a useful tool for studying NTSR mechanisms, although the transcriptomes of most weed species have not yet been fully sequenced. In recent years, RNA-Seq has been successfully applied to identify the genes involved in NTSR to the AHAS-inhibiting herbicides in two grass and one broadleaf weed species—*L. rigidum* (Gaines et al., 2014), *A. myosuroides* (Gardin et al., 2015), and *D. sophia* (Yang et al., 2016)—which generated 19,623, 180,117, and 84,085 assembled contigs with an average size of 448, 616, and 839 bp, respectively. Nevertheless, only a handful of herbicide-metabolizing and resistance-endowing genes have been identified in these weed species (Yang et al., 2016). Moreover, the NTSR mechanisms are expected to differ both among and within weed species (Délye et al., 2013). Therefore, we performed the RNA-Seq analyses twice: once for the *de novo* assembly of the transcriptome of *A. aequalis*, and again for the identification of the candidate contigs in response to the mesosulfuron-methyl in the multiple-herbicide-resistant *A. aequalis* population. Owing to the development of the Illumina Hiseq 4,000 platform, our reference *A. aequalis* transcriptome had a similar number of assembled unigenes (95 479), but of longer average size (944 bp), when compared with those reported by projects using other weed species (Peng et al., 2010; Riggins et al., 2010; Yang et al., 2013; An et al., 2014; Leslie and Baucom, 2014; Chen et al., 2016; Duhoux et al., 2017).

A commercial formulation of mesosulfuron-methyl was used in our experiments because any allele(s) that enables an individual plant to withstand herbicide action should be strongly selected by recurrent applications, of both the herbicide molecules and the formulations associated with the herbicides, under realistic field conditions (Délye, 2013). In addition, seeds of the R population were collected from Anhui and those of the S population were collected from Shandong (Guo et al., 2016). Because of the differences in the genetic background of the two populations, and also in the application of herbicides, both the constitutive and herbicide-induced DEGs whose functional annotation involved metabolic resistance between the two biotypes were selected. Subsequently, all the contigs were confirmed by qRT-PCR of the RNA-Seq samples and again by the parallel plant materials. Hence, the genes consistently over-expressed in the R plants may contribute importantly to the NTSR. The 17 genes identified here support the hypothesis that NTSR in *A. aequalis* is largely driven by the differences in the expression patterns of a set of genes between the R and S plants.

### Candidate genes in the CytP450, GST, and GT families

In our study, four CytP450 genes (*CYP94A1, CYP94A2, CYP71A4*, and *CYP734A6*) were identified as major candidates for metabolic resistance. The biochemical role of CytP450-mediated herbicide metabolism has been well-established in resistant *L. rigidum* populations, and several distinct CytP450 isoforms were shown to be involved in metabolic resistance to ACCase- and AHAS-inhibiting herbicides (Christopher et al., 1994; Preston et al., 1996; Preston and Powles, 1998; Busi et al., 2011; Duhoux and Délye, 2013; Gaines et al., 2014). Several CytP450 genes, such as *CYP71AK2* and *CYP72A254*, were reported to show a high expression level in a multiple-herbicide-resistant *E. phyllopogon* population (Iwakami et al., 2014b), while *CYP94A1* was implicated in the process of plant defense against chemical injury in the common vetch (*Vicia sativa* L.) (Benveniste et al., 2005). The *CYP734A* family typically functions in the brassinosteroid catabolism of plants, and brassinosteroids are known to protect plant crops against toxicity from herbicides (Ohnishi et al., 2006; Xia et al., 2009). Transgenic Arabidopsis (*Arabidopsis thaliana*) expressing either of the two CytP450 genes, *CYP81A12* or *CYP81A21*, confers resistance to AHAS inhibitors bensulfuron and penoxsulam (Iwakami et al., 2014a). Moreover, many CytP450s conferring herbicide resistance have been identified in several major crop species, such as *CYP81A6* in rice (*O. sativa*) (Pan et al., 2006), *CYP71C6v1* in wheat (*T. aestivum*) (Wen-Sheng et al., 2006), *CYP71A10* in soybean (*Glycine max* [Linn.] Merr.) (Siminszky et al., 1999), and *CYP76B1* in Jerusalem artichoke (*Helianthus tuberosus* L.) (Didierjean et al., 2002). Importantly, it has been demonstrated that the purified recombinant protein expressed by the *T. aestivum CYP71C6v1* gene could specifically metabolize three herbicides, namely chlorsulfuron, triasulfuron, and metsulfuron-methyl, via the phenyl ring hydroxylase (Wen-Sheng et al., 2006; Xiang et al., 2006). The CytP450 genes that we identified in the R *A. aequalis* population may also confer potential resistance to multiple herbicides, but this hypothesis will require empirical testing for its validation.

The GSTs and GTs are two other key families of enzymes involved in herbicide detoxification. The biochemical role of GST and GT in the NTSR to herbicides is now well-established (Brazier-Hicks and Edwards, 2005; Cummins et al., 2011, 2013). Prior studies indicated that GSTs and GTs were associated with NTSR to the ACCase- and AHAS-inhibiting herbicides in *L. rigidum* (Gaines et al., 2014; Duhoux et al., 2015). In the present study, two GST genes (*GST-T3* and *GST-F1*) and two GT genes (*GT83A1* and *GT75D1*) were identified as associated with the metabolic resistance of *A. aequalis*. The predicted *GST-F1* gene shows a high sequence homology with *A. myosuroides* (86%, UniProt accession No. Q9ZS17). Another study showed *GST-F1* plays a central role in the multiple-herbicide resistance of *A. myosuroides*, and that the expression of its orthologous *GST-F1* in a multiple-resistant *L. rigidum* population also showed enhancement (Cummins et al., 2013). Both the Tau (U) and Phi (F) classes of GSTs play roles in the herbicide detoxification performed by plants for several compounds (Cummins et al., 2011). Some GST genes conferring herbicide tolerance were also identified in several crop species, such as *TaGSTU4* in wheat (*T. aestivum*) (Thom et al., 2002), *GST I*, and *GST-27* in maize (*Zea mays* L.) (Karavangeli et al., 2005), and *GmGSTU21* and *GmhGS* in soybean (*G. max*) (Skipsey et al., 2005). Additionally, the *GT75D1* found in the present study shares a 73% sequence identity with the predicted 5-O-glucosyltransferase gene of *B. distachyon* (GenBank accession No. XP_003571494.1). Coincidentally, an *A. myosuroides* population, one resistant to multiple classes of herbicides, due in part to the elevated expression of CytP450s and GSTs active in herbicide detoxification, displayed higher O-glucosyltransferase activity than did the herbicide-sensitive populations of *A. myosuroides* (Brazier et al., 2002). Similarly, GT gene activities, for example *GTA* triggered by the application of AHAS-inhibiting herbicides, were observed in *A. thaliana* (Manabe et al., 2007), and two GT genes were shown to glycosylate the primary major bentazone metabolite, 6-hydroxybentazone, in the herbicide-tolerant *G. max* (Leah et al., 1992). Together, these findings suggest to us that the *GST-F1* and *GT75D1* genes up-regulated in the R *A. aequali* population may be associated with its mesosulfuron-methyl resistance. However, the respective roles of *GST-T3* and *GT83A1* remain unknown, and more investigative work is needed to elucidate their putative roles in herbicide resistance.

### Candidate genes in the ABC transporter family

Plant ABC transporters, one of the most diverse gene families, are implicated in the detoxification of xenobiotics, which include herbicides (Rea, 2007). However, in contrast to the CytP450, GST, and GT gene families which confer herbicide resistance through metabolism, the ABC transporters detoxify herbicides by sequestering the herbicides and their metabolites (Yuan et al., 2007). In the present study, the two contigs c34002_g1 and c39076_g1 were annotated to *ABCC8* and *ABCB11*, respectively, and so they may be associated with the mesosulfuron-methyl resistance. Similar to our result, an *ABCC1*, which could play an important role in the metabolic resistance to tribenuron-methyl, was identified in the resistant *D. sophia* population (Yang et al., 2016); an *ABCB11* was also identified and found up-regulated in a resistant *B. syzigachne* population having NTSR to fenoxaprop-*P*-ethyl (Pan et al., 2016). Indeed, there are considerable lines of evidence for the involvement of ABC transporters in protecting plant tissues from toxin-induced damage. Two ABCC transporters, *AtABCC1* and *AtABCC2*, were reported to mediate the tolerance of arsenic and arsenic-based herbicides in *A. thaliana* (Song et al., 2010). An ABC transporter, *AtOPT6*, showed an association with herbicide resistance in *A. thaliana* (Cagnac et al., 2004). Finally, the overexpression of *AtPgp1*, a multidrug resistance family member, and that of its garden pea homolog *psNTP9*, was able to confer resistance to multiple herbicides in *A. thaliana* (Windsor et al., 2003). Therefore, it is likely that *ABCC8* and *ABCB11* participate in driving metabolic resistance to mesosulfuron-methyl.

### Candidate herbicide-resistance genes in other gene families

In this study, we found five further genes possibly associated with mesosulfuron-methyl metabolic resistance in *A. aequalis*. These genes are annotated in three major enzyme families functioning in the herbicide degradation process: hydrolases, oxidases, and peroxidases. Hydrolases can cleave the herbicide molecule, after which the cleaved molecule is transformed into a more hydrophilic metabolite by the oxidases and peroxidases (Délye, 2013). In addition, the increased expression of oxidases and peroxidases could effectively protect plant cells against the oxidative damage caused by a herbicide's action, which may also contribute to herbicide resistance (Délye, 2013).

Additionally, we selected two up-regulated contigs potentially encoding proteins with homology to kinase involved in response to the stimulus of salicylic acid (a plant hormone). Salicylic acid is a signaling molecule involved with local plant defense reactions at infection sites and the induction of systemic resistance (Durner et al., 1997). Plants can withstand various biotic and abiotic stresses in variable environments by having evolved complicated networks of stress detection, signaling, and response pathways which trigger both general and specific responses, often with an adjustment of the response over time (Délye, 2013). As a powerful abiotic stress, herbicides are known to trigger some of these pathways depending on the specific type of study, the herbicide, and the plant species involved (Das et al., 2010; Unver et al., 2010). NTSR is clearly part of an abiotic stress response, but the extent of this involvement varies among genotypes. Importantly, hormones participate in optimizing plant responses to abiotic stresses (Peleg and Blumwald, 2011), and yet there are few reports of such responses involving NTSR. The up-regulated genes annotated as hormone-related in this study may well be related to the mesosulfuron-methyl-induced stress response pathways. Considering the complexity of most plant defense mechanisms, further rigorous and comprehensive studies are required.

### Optimization of methods

In this study, the RNA-Seq experimental design consisted of three treatments: an untreated control, a water-only control, and a mesosulfuron-methyl treatment. The objectives of using two controls were to measure the effects on transcription due specifically to the mesosulfuron-methyl treatment, and to control for any transcriptional effects from the spraying of plants with water (the solvent for mesosulfuron-methyl). According to our results, more DEGs were evidently observed due to mesosulfuron-methyl treatment relative to the untreated control than due to mesosulfuron-methyl treatment relative to the water-only control (Figure 7A). Hence, the DEGs triggered specially by mesosulfuron-methyl may play a more important role in the NTSR of *A. aequalis*. An earlier RNA-Seq experiment likewise established two controls—an untreated control and adjuvant-only control—to eliminate the potential effects on transcription due to the adjuvant (Gaines et al., 2014). By contrast, many other published reports pay more attention to the comparison between the untreated control and the herbicide treatment, which neglects the possible effects of the solvent or adjuvant on the RNA-Seq results (e.g., Yang et al., 2013, 2016; An et al., 2014; Chen et al., 2016).

## Conclusion

A multiple-resistant *A. aequalis* population (AH18) possessing TSR and NTSR to mesosulfuron-methyl and fenoxaprop-*P*-ethyl was identified. The TSR mechanism was clearly demonstrated in our previous work, while the NTSR mechanism was explored in the current study. Taken together, the results indicate this latter mechanism is likely due to CytP450-, GST-, and GT-mediated integrated metabolic resistance, in combination with an ABC transporter-mediated sequestration of the herbicide metabolites. Additional genes, whose functional annotation is related to oxidation, hydrolysis, and even plant stress physiology, may also contribute to the NTSR. All the candidate genes identified in this study were triggered by mesosulfuron-methyl, and so it may also be responsible for the NTSR to fenoxaprop-*P*-ethyl. The current study is but a first step toward elucidating the mechanism behind NTSR in *A. aequalis*. Functional characterizations, ideally including transgenic expression and over-expression in a model plant species, *B. distachyon*, and key-gene knockouts in the R *A. aequalis* population, are now necessary to define the biochemical role of the candidate genes in herbicide metabolism and resistance in plants.

## Data accessibility

The raw Illumina sequence reads have been deposited in the NCBI Sequence Read Archive (SRA) database with accession number SRP106664 including SRX2792093 for SD01 (S) and SRX2792094 for AH18 (R).

## Author contributions

NZ and JW designed the research. NZ, WL, and SB performed the experimental work and the data analysis. WG, GY, FW, and WTL provided helpful suggestions for the data analysis and during the manuscript preparation. NZ and JW wrote the paper. All authors edited and reviewed the manuscript.

### Conflict of interest statement

The authors declare that the research was conducted in the absence of any commercial or financial relationships that could be construed as a potential conflict of interest.
