# Supplementary material for: Transcriptome Profiling to Identify Genes Involved in Mesosulfuron-Methyl Resistance in Alopecurus aequalis
Source: Front Plant Sci. 2017 Aug 9;8:1391. doi: 10.3389/fpls.2017.01391 (PMC5552757; doi:10.3389/fpls.2017.01391)
Supplement: Supplementary file 2 [file Table2.DOCX]

**Supplementary Table S2. The respective numbers of overlapping up-regulated genes in all three comparative treatment groups: R_T relative to S_T, R_T relative to R_WCK, and R_T relative to R_CK, with their corresponding predicted annotations as related to the P450s, GSTs, GTs, and ABC transporters.** ^a^Number of common up-regulated genes between the comparative groups of R_T relative to S_T and R_T relative to R_WCK. ^b^Number of common up-regulated genes in all three comparative treatment groups.

| Annotated classification | Number of up-regulated genes in the comparable group | | | |  |
| --- | --- | --- | --- | --- | --- |
|  | R_T vs. S_T | R_T vs. R_WCK | In common^a^ | R_T vs. R_CK | In common^b^ |
| P450s | 104 | 37 | 10 | 39 | 5 |
| GSTs | 22 | 9 | 1 | 9 | 1 |
| GTs | 71 | 31 | 4 | 39 | 2 |
| ABC transporters | 37 | 18 | 2 | 24 | 1 |
