# Supplementary material for: Transcriptome Profiling to Identify Genes Involved in Mesosulfuron-Methyl Resistance in Alopecurus aequalis
Source: Front Plant Sci. 2017 Aug 9;8:1391. doi: 10.3389/fpls.2017.01391 (PMC5552757; doi:10.3389/fpls.2017.01391)
Supplement: Supplementary file 3 [file Table3.DOCX]

**Supplementary** **Table S3. The respective numbers of overlapping up-regulated genes with significantly different levels of expression between the R and S populations in all three comparative treatment groups, annotated as the P450s, GSTs, GTs, and ABC transporters.** ^a^Number of overlapping up-regulated genes in all three comparative groups of R_T relative to S_T, R_WCK relative to S_WCK, and R_CK relative to S_CK. ^b^HE, higher expression: The number of overlapping genes whose |log2(fold change)| value in R_T relative to S_T exceeded that of the other two comparative groups (i.e., R_WCK relative to S_WCK, and R_CK relative to S_CK).

| Annotated classification | Number of up-regulated genes in the comparable group | | | | | | | |
| --- | --- | --- | --- | --- | --- | --- | --- | --- |
|  | R_T relative to S_T | R_WCK relative to S_WCK | R_CK relative to S_CK |  | In common^a^ |  | HE^b^ |  |
| P450s | 104 | 94 | 52 |  | 28 |  | 3 |  |
| GSTs | 22 | 30 | 15 |  | 8 |  | 4 |  |
| GTs | 71 | 65 | 47 |  | 21 |  | 3 |  |
| ABC transporters | 37 | 55 | 21 |  | 9 |  | 1 |  |
