# Supplementary material for: Transcriptome Profiling to Identify Genes Involved in Mesosulfuron-Methyl Resistance in Alopecurus aequalis
Source: Front Plant Sci. 2017 Aug 9;8:1391. doi: 10.3389/fpls.2017.01391 (PMC5552757; doi:10.3389/fpls.2017.01391)
Supplement: Supplementary file 4 [file Table4.DOCX]

**Supplementary Table S4. Primer sequences used for the qRT-PCR relative quantification of gene expression in *Alopecurus aequalis*.**

| Gene ID | Fwd/Rev | Function annotation | Sequence (5' to 3') |
| --- | --- | --- | --- |
| c42830_g1 | fwd | CYP71C2 | TTCATCGTCTTCGCTGG |
|  | rev |  | GAAAGCAAGGGGAAGGACT |
| c39857_g2 | fwd | CYP94A2 | CAAGAGCCTTCAGTACACCCA |
|  | rev |  | GAACGCCTCGTAGTCGGT |
| c46166_g6 | fwd | CYP71D10 | TCCCAAACTTCTCGCTCATGT |
|  | rev |  | TCGTGCTGGCTAATCTCCTGT |
| c50769_g1 | fwd | CYP71D8 | CCCTCGCCGACCTGAACTA |
|  | rev |  | TGTCACCACGCTCGAATCTTT |
| c21190_g1 | fwd | CYP94A1 | GCAAGCCAAAGAAACAACAGAG |
|  | rev |  | CGAGGAGCCAGAAGAACCAC |
| c43350_g3 | fwd | CYP71A4 | CGTCACGAGAAACGGAATGGT |
|  | rev |  | GTCAAAGATGGCGATCAGGG |
| c45454_g1 | fwd | CYP734A6 | GTCGTTTCCGTAGCCCAT |
|  | rev |  | CCAAATCCCAGCACTTAGGT |
| c46602_g8 | fwd | CYP86B1 | GCTGCCTCATTGCTTTCCA |
|  | rev |  | TGCTCGACCTGACCACCCT |
| c21481_g1 | fwd | GST-T3 | TGTTGGGTATGCCCTTAGGG |
|  | rev |  | AGCAGATGCGGGAAGGGTAG |
| c46293_g5 | fwd | GST-T3 | CTGTTGTTTCAGTAACCTCGTCCC |
|  | rev |  | GCTAAAGTTGCGGCTTCCATT |
| c42028_g1 | fwd | GST-Z2 | TGAGCGACAAACAAGCGAAGT |
|  | rev |  | CCTCAGCAAGTGGAGCGATA |
| c35468_g1 | fwd | GST-Z2 | AGAGGAGCGAAGATAGATAGACGA |
|  | rev |  | TGCATGATTCCACCCAAGAC |
| c45520_g1 | fwd | GST-F1 | GACGGCGGACTCGCAGAT |
|  | rev |  | AGCATCCGAAGCAGGCAG |
| c49096_g1 | fwd | GT92A1 | TACGGGAGCGTTCCTTAGCC |
|  | rev |  | GGCCAGCATCTTGACGTTGTA |
| c44063_g7 | fwd | GT75D1 | CTCCTTCTGCCGCCTGCT |
|  | rev |  | AGCCGTCCTATCGCTCTTGG |
| c26389_g1 | fwd | GT83A1 | GCTTCGTGTCCCACTGCG |
|  | rev |  | GCAGATGTAGCTCCGGTTCAG |
| c45451_g12 | fwd | GT73B5 | CGCAGGTATGCCCTTAGTGG |
|  | rev |  | GGAGCTTCGGCGAGGTGT |
| c26062_g1 | fwd | GT73C5 | GAGCTCCCTTGCGATGTCAC |
|  | rev |  | TACTGGACGGGTGCGAGAAC |
| c34002_g1 | fwd | ABCC8 | GTGGCGGCAACCATAGTGA |
|  | rev |  | GCGTCCGTGTCGATGAGCT |
| c39076_g1 | fwd | ABCB11 | TTAGCAGGTAACCGCATTCAC |
|  | rev |  | CCCTAACGGATGTTTCTTTCA |
| c38775_g3 | fwd | Peroxidase | GGCTGGATGCTGAGGTTCTG |
|  | rev |  | CTCCGCATCTTCTTCCACGA |
| c38555_g1 | fwd | NADH Oxidase | GGCTTCAACCTGCTCCTGC |
|  | rev |  | GACTCGGTGCCGCTCATAC |
| c33496_g2 | fwd | NADH Oxidase | CAGGATTCGCTAGGAAGAGCC |
|  | rev |  | CCAAAGCGGTTGCTGCCATAC |
| c33496_g1 | fwd | NADH Oxidase | CATACTCGTCGGTGCGGTCAT |
|  | rev |  | AGCATGTCGAGGCATGGAAGC |
| c41309_g5 | fwd | Carboxylesterase | GCTGACCGATTTGGCAACACC |
|  | rev |  | CCTCTGTTTCAGCGTGCCATC |
| c43601_g1 | fwd | Phospholipase | CGCAGAGGCGTTGTTCCAGTTAG |
|  | rev |  | TGTCGTCTGAGCCGTCTGATGTT |
| c48254_g2 | fwd | Hydrolase | TCAAGCAGATAGCGACCGACCAA |
|  | rev |  | TAGTGTCACAGGCATCCGTCCAG |
| c36906_g1 | fwd | Decarboxylase | CAGGTCCTTCACCAACAATCTT |
|  | rev |  | AAACGTCGAACCAAAAGCG |
| c39903_g1 | fwd | Hydrolase | TGAGAACCTCGGTAATCTGCTGT |
|  | rev |  | GAATGTGCTTCAGAACGGGG |
| c46657_g2 | fwd | Kinase | GGGGGGTGATCCTCGCTCTTTTA |
|  | rev |  | CGGCGTTGCTCTCCTCTCCTTAT |
| c47808_g1 | fwd | Kinase | CATAAGCTGGTCTGGGTTAAATCAA |
|  | rev |  | TGTGGCAGTTAACTAGCACCTATGG |
| Reference Genes | |  |  |
| Gene name | Fwd/Rev |  | Sequence (5' to 3') |
| UBQ | fwd |  | AAGACCTACACCAAGCCCAAG |
|  | rev |  | CAGTAGTGGCGGTCGAAGTG |
| GAPDH | fwd |  | GTATTGTTGAGGGACTGATGACC |
|  | rev |  | AGTAAGCTTGCCATTGAACTCAG |
