# Supplementary material for: Transcriptome Profiling to Identify Genes Involved in Mesosulfuron-Methyl Resistance in Alopecurus aequalis
Source: Front Plant Sci. 2017 Aug 9;8:1391. doi: 10.3389/fpls.2017.01391 (PMC5552757; doi:10.3389/fpls.2017.01391)

Supplementary Figure S1. Gene ontology (GO) enrichment analysis of the differentially expressed genes (DEGs) between the mesosulfuron-methyl-treated R (AH18) and S (SD01) populations of *Alopecurus aequalis*.

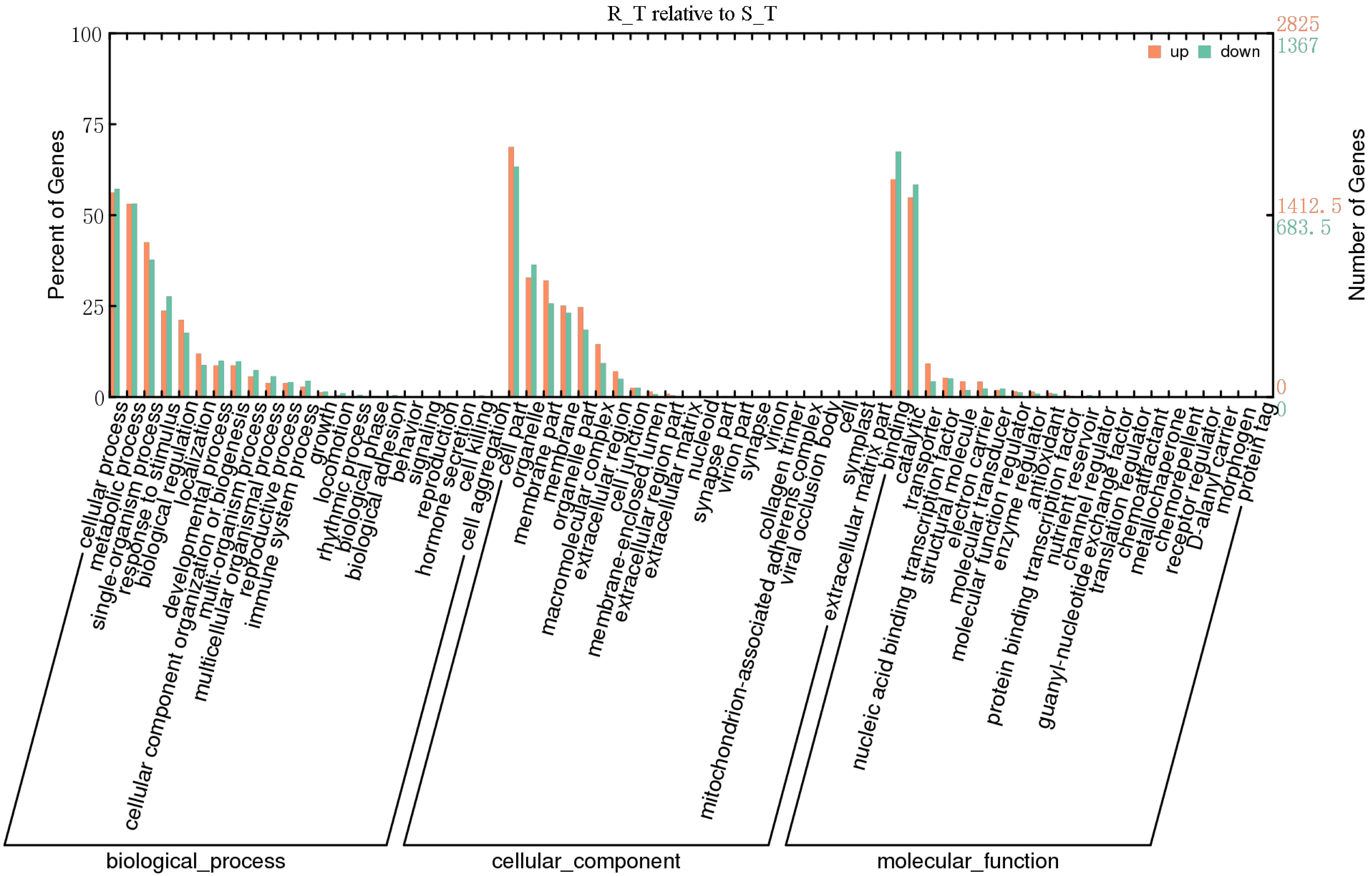

Supplement: Supplementary file 5 [file Image1.PDF]
