# Supplementary material for: Transcriptome Profiling to Identify Genes Involved in Mesosulfuron-Methyl Resistance in Alopecurus aequalis
Source: Front Plant Sci. 2017 Aug 9;8:1391. doi: 10.3389/fpls.2017.01391 (PMC5552757; doi:10.3389/fpls.2017.01391)
Supplement: Supplementary file 7 [file Image3.pdf]

Supplementary Figure S3. KEGG category enrichment of the differentially expressed genes (DEGs) between the water-treated R and S populations of *Alopecurus aequalis*. The number of genes in each category is proportional to the size of the dot. The dot color represents the *q*-value.

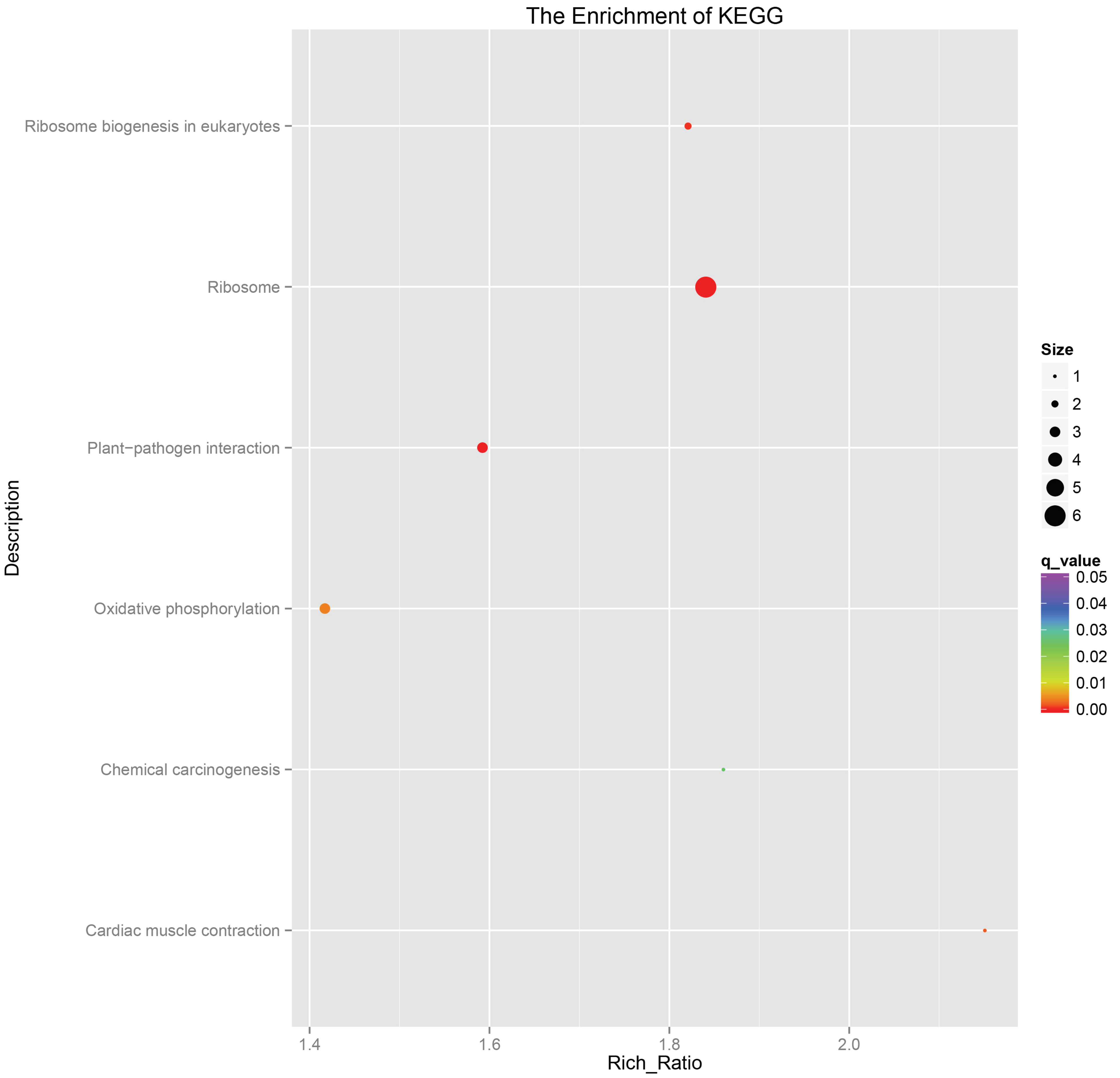

R_WCK vs. S_WCK
