# Supplementary material for: Transcriptome Profiling to Identify Genes Involved in Mesosulfuron-Methyl Resistance in Alopecurus aequalis
Source: Front Plant Sci. 2017 Aug 9;8:1391. doi: 10.3389/fpls.2017.01391 (PMC5552757; doi:10.3389/fpls.2017.01391)

Supplementary Figure S5. The qRT-PCR profiles of the 14 remaining genes that did not consistently show up-regulated expression in the R *Alopecurus aequalis* samples. Blue bars represent the RNA-Seq; red bars represent the qRT-PCR validation that used the RNA-Seq samples; and green bars represent the qRT-PCR validation that used the parallel plant materials. Both *UBQ* and *GAPDH* were used as the internal control genes. Means and their SEs from three biological replicates are shown.

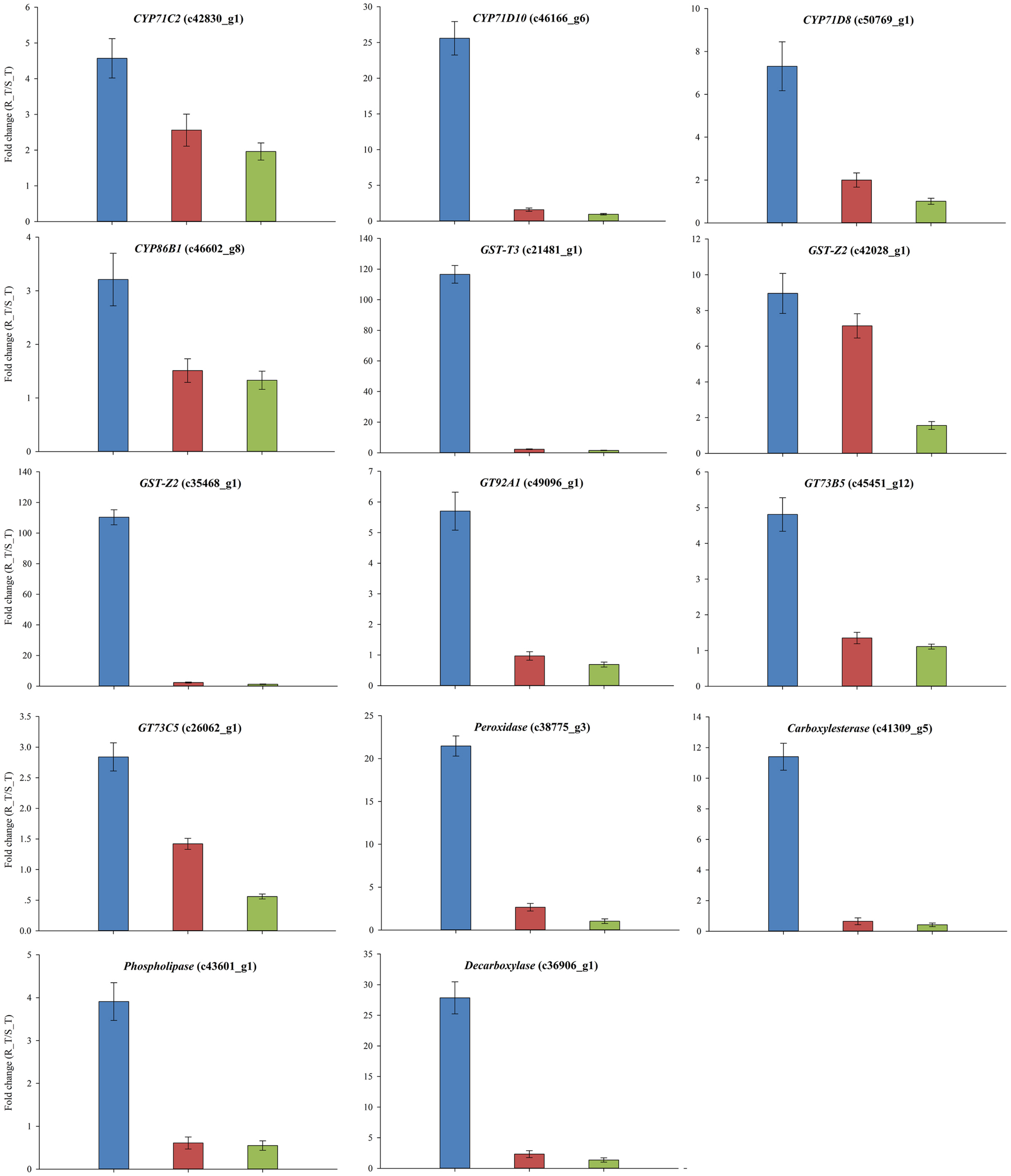

Supplement: Supplementary file 9 [file Image5.PDF]
